# Acute Kidney Injury (AKI) before and after Kidney Transplantation: Causes, Medical Approach, and Implications for the Long-Term Outcomes

**DOI:** 10.3390/jcm10071484

**Published:** 2021-04-02

**Authors:** Alessandra Palmisano, Ilaria Gandolfini, Marco Delsante, Chiara Cantarelli, Enrico Fiaccadori, Paolo Cravedi, Umberto Maggiore

**Affiliations:** 1 Department of Medicine and Surgery, University of Parma, 43126 Parma, Italy; ilaria.gandolfini@gmail.com (I.G.); delsantem@gmail.com (M.D.); chiaracantar@gmail.com (C.C.); enrico.fiaccadori@unipr.it (E.F.); umberto.maggiore@unipr.it (U.M.); 2 Nephrology Unit, Parma University Hospital, 43126 Parma, Italy; 3 Renal Division, Department of Medicine, Icahn School of Medicine at Mount Sinai, New York, NY 10029, USA; paolo.cravedi@mssm.edu

**Keywords:** acute kidney injury, kidney transplantation, delayed graft function, donor selection

## Abstract

Acute kidney injury (AKI) is a common finding in kidney donors and recipients. AKI in kidney donor, which increases the risk of delayed graft function (DGF), may not by itself jeopardize the short- and long-term outcome of transplantation. However, some forms of AKI may induce graft rejection, fibrosis, and eventually graft dysfunction. Therefore, various strategies have been proposed to identify conditions at highest risk of AKI-induced DGF, that can be treated by targeting the donor, the recipient, or even the graft itself with the use of perfusion machines. AKI that occurs early post-transplant after a period of initial recovery of graft function may reflect serious and often occult systemic complications that may require prompt intervention to prevent graft loss. AKI that develops long after transplantation is often related to nephrotoxic drug reactions. In symptomatic patients, AKI is usually associated with various systemic medical complications and could represent a risk of mortality. Electronic systems have been developed to alert transplant physicians that AKI has occurred in a transplant recipient during long-term outpatient follow-up. Herein, we will review most recent understandings of pathophysiology, diagnosis, therapeutic approach, and short- and long-term consequences of AKI occurring in both the donor and in the kidney transplant recipient.

## 1. Introduction

Acute kidney injury (AKI), a common problem in kidney transplantation, can take place both in the donor before organ harvesting, and in the recipient early after transplantation. It manifests as delayed graft function (DGF) or de novo post-transplant acute deterioration of graft function. In either setting, AKI in the graft could affect short- and long-term transplant outcomes.

DGF, which is a heterogeneous condition resulting from factors related to procurement, organ quality, recipient medical condition, surgical insult, and graft injury-related to dialysis treatment itself, is most commonly defined as the requirement of dialysis sessions in the first week of post-transplantation in a patient who eventually becomes free of dialysis [1]. However, whereas the definition of AKI has been standardized, the need for dialysis (which defines DGF) has not, and therefore the designation DGF is inaccurate.

According to recently published data, AKI affects 30% of kidneys coming from deceased donors and 50% of those coming from deceased donors after cardiac death (DCD) [2,3]. Although DGF increases the risk of acute cellular rejection and reduces graft survival [4], to date, there is no Food and Drug Administration (FDA) approved therapy for DGF, although several treatment strategies have been proposed, and numerous clinical trials are ongoing.

AKI may develop post-transplantation after an initial recovery of kidney function or may occur late after transplantation. In both settings, AKI can originate from severe and often unrecognized clinical conditions that may require a prompt intervention to prevent graft loss.

The present review focuses on the etiology, diagnosis, prognosis, and treatment of AKI in both the donor and the recipient, both in the short and long term.

### 1.1. Acute Kidney Injury in the Donor DGF and Risk of Graft Failure

The organ shortage is causing an ever-increasing gap between the availability of organs and transplant candidates, therefore the use of less than optimal donor kidneys, like organs from expanded criteria donors (ECD), or donors after cardiac death, has augmented over the last two decades in order to expand the deceased-donor pool [5]. These policies have been developed under the assumption that a survival benefit over dialysis can be achieved in most patients even with the lowest quality donors [6,7,8,9]. The use of donors with AKI adds to these strategies for expanding the deceased-donor pool.

AKI, which occurs in more than 25% of critically ill patients, depends on the underlying disease, the duration of kidney impairment, and the patient’s baseline kidney condition [10]. From experiences outside the context of transplantation, it is well known that, provided that the patient baseline kidney function is normal, ischemic or toxic insults causing AKI do not generally hamper full recovery of kidney function [11,12]. On this basis, it is widely accepted that kidneys from donors with AKI might represent a suitable and safe source for kidney transplantation. Nonetheless, in many European national or international organ-sharing systems, it is current practice to decline about 50% of these kidneys with severe AKI (i.e., Kidney Disease Improving Global Outcomes (KDIGO) stage 2 and 3) due to a perceived higher risk of poor outcome after transplantation [13]. The same scenario applies to the United States, where more than 18% of the kidneys recovered for transplant are discarded, and donor AKI more than doubles the discard rate [14].

Several studies have been published to assess the effect of donor AKI on primary no function, DGF, and long-term graft dysfunction. By and large, those studies showed that donor AKI might cause DGF but not necessarily increase the risk of early graft loss or graft failure in the long term.

Kayler et al. [15], by analyzing registry US data from Scientific Registry of Transplant Recipients during the period 1995–2007, found that kidneys from donors with a terminal creatinine ≥ 2.0 mg/dL were associated with an increased risk of allograft failure only if they were procured from ECD, the relative increased risk of allograft failure in this setting being +17%.

A subsequent retrospective study [13] analyzed the outcome of approximately 12,000 donors (72% of the offered donors) from the UK Transplant Registry (2003–2013). The study assessed graft function/primary nonfunction (PNF), estimated glomerular filtration rate (eGFR), and graft-survival at 90 days and 1 year were evaluated. Approximately 1900 donors (17%) were classified as AKI stage 1–3, according to AKI Network (AKIN) criteria. Graft failure at 1 year was greater for donors with AKI than for those without (graft survival 89% vs. 91%; DGF rates increased with donor AKI stage; *p* < 0.005), and PNF rates were significantly higher for AKIN stage 3 kidneys (9% vs. 4%, *p* = 0.04) [13].

In a US study from five organ procurement organizations in the period 2010–2013, Hall et al. [14] reported a more reassuring scenario. Even though in this study DGF rate progressively increased from 28% for kidneys from donors without AKI to 34%, 52%, and 57% for donor AKI stage 1, 2, and 3 respectively, eGFR at six months post-transplantation was well-preserved irrespective of donor AKI stage [14]. However, two thirds of procured kidneys from donors with AKI stage 3 underwent machine perfusion, and approximately 90% underwent procurement biopsy to evaluate kidney quality histologically. A longer follow-up study was more recently reported by the same authors [16], including a total of 2430 transplants, of which 585 (24%) were from donors with AKI. None of the donors required dialysis. Although there was an early increase in graft loss among recipients of donors with AKI stage 3, the study confirmed that deceased-donor AKI was not associated with kidney allograft failure in the long term. In fact, after a median follow-up period of 4.0 years (interquartile range, 3.0–5.0 years), there were 623 (26%) all-cause graft failures, which included 402 deaths and 313 death-censored graft failures: all-cause graft failure was not statistically different across different AKI stages [16]. By comparing donors with and without AKI, donors with AKI were older, had a higher mean kidney donor profile index (KDPI), had a longer mean cold ischemia time (CIT), and were more likely to undergo machine perfusion. Rather surprisingly, the presence of unfavorable donor-related risk factors such as high KDPI and long CIT (>14 h) did not affect the relationship between deceased-donor AKI and graft loss. The authors concluded that the current practice of using kidneys from donors with AKI does not adversely affect post-transplant outcomes beyond the first year, despite the increased risk of early complications (DGF).

Since both donor AKI and prolonged CIT are associated with an increased risk of DGF, Dube et al. [17] tested the impact of the synergic effect of prolonged CIT and donor AKI on allograft survival. By using data from the United Network for Organ Sharing (UNOS), the authors analyzed all deceased donor kidney transplantations (years 2005–2015) that had CIT > 24 h and AKI (terminal serum creatinine ≥ 2 mg/dL). The study included 8071 recipients of kidneys from a donor with AKI: 5434 kidneys with CIT < 24 h, 1289 kidneys with 24–30 h of CIT, 734 kidneys with 30–36 h of CIT, and 614 kidneys with >36 h of CIT. The median KDPI as well as the proportion of ECD and high KDPI kidneys was slightly lower in the group with <24 h of CIT. This study first confirmed the increasing tendency of using kidneys from donors with AKI and prolonged CIT (approximately 1 of every 30 donors in the United States came from a donor with AKI and CIT greater than 24 h); secondly, the study showed that DGF rate was 43.8% in the total cohort and that DGF increased with CIT above 24 h. Accordingly, death-censored graft survival at 3 years was better with CIT < 24 h compared with other groups (92.5% vs. 90.8% vs. 92% vs. 89.2%, in the CIT < 24, 24–30, 30–36, and 36+, respectively).

A recent mono-centric study examined the long-term outcomes of a large cohort of kidney transplants from donors with AKI, in which pre-specified criteria for acceptance were based on pre-implantation biopsy [18]. Kidneys with AKI were accepted unless they had >10% cortical necrosis or more than mild chronic changes. The cohort included 1313 kidneys from 974 donors, AKIN stage 0 (no AKI) in 319 (24.3%), stage 1 in 370 (28.2%), stage 2 in 177 (13.5), and stage 3 in 447 (34.0%). Estimated 5-year graft survival (95% confidence interval) was 78.5% (72.5–84.5), 77.8% (72.8–82.1), 83.8% (76.8–88.9), and 84.6% (79.5–88.7) for AKIN donor stage 0 to 3, respectively (log-rank *p* = 0.10). In this setting of biopsy-driven acceptance criteria, the severity of deceased-donor AKI did not affect the relation between DGF and graft loss [18].

In conclusion, current evidence supports the notion that, for standard-risk donors, donor AKI does not impair transplantation outcomes. The evidence is less convincing for marginal donors, such as elderly donors or donors with elevated KDPI (e.g., above 85%). For the latter type of donors, we would suggest that protocols for organ quality assessment, minimization of cold ischemia times, and/or use of machine perfusion be implemented at each transplant center.

### 1.2. Recipient with AKI Early Post-Transplantation

As outlined above, DGF may have an unfavorable impact on allograft outcomes, including long-term kidney allograft function, and on patient and graft survival. This unfavorable association is modified by the severity of DGF, as indicated by the duration of dialysis dependence after transplant: the longer the dialysis-dependent period, the higher the hazards of rejection and of graft failure [19,20]. The relationship between the duration of dialysis-dependent period and death-censored graft failure, and also the proportion of death-censored graft failure that is mediated by DGF-induced acute rejection, was investigated by Lim et al. using data from the Australian and New Zealand Dialysis and Transplant Registry [21]: of 7668 deceased donor kidney transplants (years 1997–2014), 19.5% of the recipients had DGF. The median (interquartile range) duration of DGF was 7 (9) days, with 25% requiring dialysis for 14 days or longer. Compared to recipients who had experienced a DGF duration of 1 to 4 days, the adjusted hazard ratios associated with duration of 5 to 7, 8 to 13, and 14 days or longer were 1.13, 1.44, and 1.99 respectively, for acute rejection, and were 1.10, 1.45, and 1.60 respectively, for death-censored graft loss. There was a direct dose-dependent effect between DGF duration and death-censored graft loss, with DGF beyond 7 days post-transplant being associated with a more than 40% greater risk of death-censored graft loss. By using mediation analysis, they showed that the proportion of death-censored graft loss that is mediated by DGF-induced acute rejection is less than 10%. These findings support the notion that other mechanistic pathways, besides acute rejection, may mediate the relation between DGF and accelerated long-term graft loss.

As mentioned above, DGF recognizes a multifactorial pathogenesis involving donor-related risk factors, recipient-related risk factors, and perioperative risk factors (Table 1) [22,23,24]. Donor-related risk factors included variables related to peri-donation time, such as the presence of AKI before donation, hemodynamic instability in ICU requiring vasopressor use, and prolonged CIT. Graft quality has been recognized as an important determinant for DGF: old age, presence of chronic kidney disease (CKD) risks factors (e.g., hypertension, diabetes), and high kidney donor profile index might in fact predict insurgence of DGF [25]. Finally, it is well established that DGF depends on donor type being more frequent with DCD than DBD (donation after brain death) donors and almost absent with the use of living donors.

Recipient-related risk factors are due in part to pre-transplantation variables and in part to surgery. Pre-transplantation oliguria, especially in patients with long dialysis vintage, is an important risk factor for DGF as well as the need of pre-surgery hemodialysis sessions with ultrafiltration. In addition, high immunological risk profile (e.g., hyperimmune patients, history of preview transplant) is an independent predictor of DGF related to immunosuppressive therapy, mainly due to the requirement of high dosage of calcineurin inhibitor (CNI) [26].

Moreover, a complex vascular surgery or the occurrence of vascular complications implicates a prolonged warm ischemia time, defined as the time from organ removal from cold storage to allograft reperfusion. Also, concomitant surgery (e.g., adult dominant polycystic kidney disease nephrectomy) and increased body mass index (BMI) may significantly prolong warm ischemia time [27].

Finally, perioperative factors such as post-transplant hypotension and hypovolemia and high CNI blood levels may increase the risk of DGF.

Most of the above-mentioned risk factors for DGF act by promoting ischemia-reperfusion injury. The molecular and cellular events that occur in IRI are complex, involving oxidative damage and the activation of the innate immune system [28]. Signaling components of particular relevance are the pathways initiated by the activation of Toll-like receptors (TLRs), sphingosine-1-phosphate (S1P) receptors, and hypoxia-inducible factors (HIF Toll-like receptors), activated by exogenous and endogenous ligands in response to external and internal stresses (e.g., trauma, ischemia, surgery, reperfusion) [29]. In fact, damage-associated molecular patterns (DAMP) and pathogen-associated molecular patterns (PAMP) released during ischemic injury can activate innate and adaptive immune system. The signaling causes numerous downstream effects, such as production of pro-inflammatory cytokines (e.g., IL-1, IL-6, IL-8, TNF-α), chemokines, promoting chemotaxis, opsonization, and activation of leucocytes like macrophages, neutrophils, and natural killer cells. Those mechanisms eventually cause activation of cell death programs (apoptosis and necrosis), endothelial dysfunction, loss of specific phenotype of endothelial cells, and transmigration of leucocytes into the interstitial space. Additionally, immature dendritic cells are able to activate the adaptive immune system in a direct manner by antigen presentation to T-cells or indirectly via cytokine signaling. This leads to favor T-cell-mediated rejection as well as anti-body-mediated rejection [30]. All these processes might eventually contribute to the development of interstitial fibrosis and tubular atrophy [31].

Formation of graft fibrosis is the most likely mediator of the relationship between DGF and reduced long-term graft survival. Ischemia may also induce graft fibrosis by mechanisms that do not involve inflammatory injury. For instance, one study has shown that DNA hypermethylation of kidney transplants after ischemia is directly proportional to the duration of cold ischemia [32]. In that study, genome-wide DNA methylation was profiled in three cohorts of brain-dead donor kidney allograft biopsy specimens: specimens were obtained at allograft procurement (pre-ischemia; *n* = 13), after implantation and reperfusion (postischemia; *n* = 13), and at 3 or 12 months after transplant (*n* = 5 each). Methylation increased drastically in all allografts as a result of graft ischemia. Graft ischemia preferentially reduced the expression of genes involved in suppressing kidney injury and fibrosis in terms of glomerulosclerosis and interstitial fibrosis [32]. DNA methylation modifications have been also shown to accelerate kidney aging [33]. Interestingly, hypermethylation of klotho promoter, the principal anti-aging and reno-protective factor, reduces Klotho gene expression and increases the risk of CKD severity [34]. Klotho is however also involved in the inflammatory injury. Complement modulation of klotho was found to contribute to DGF-associated chronic allograft disfunction. On the other hand, more recently, several studies showed that complement factors might directly trigger fibrogenesis, by inducing tubular apoptosis, endothelial-to-mesenchymal transition, pericytes-to-mesenchymal transition, and accelerated senescence [35,36,37,38].

Unfortunately, despite the new advances in knowledge of the mechanisms that link DGF to long term outcomes, evidence of benefit of newer strategies for diagnosis and management of AKI are still lacking.

### 1.3. Biomarkers of DGF

Monitoring of DGF in transplanted kidneys has been traditionally based on a combination of clinical (e.g., serum creatinine, urinary output), immunological (e.g., donor-specific antibodies, DSA), instrumental (e.g., resistive index at Doppler ultrasound), and histological parameters. Because of the limits and the complexity of the “traditional biomarkers”, over the last decade, new biomarkers have been introduced that can be easily measured in biological fluids, such as perfusion solution, patient’s serum, plasma, or urine [37]. Among them, those that can be measured in graft preservation fluid or in the perfusate of machine-perfused kidneys, have been proposed for organ allocation [39], and those that are measured in the recipient at the time of transplantation have been proposed to predict the occurrence of DGF and graft function recovery. However, to date, no biomarker has been sufficiently validated to be recommended for routine decision-making purposes at individual level.

Among the most promising predictive donor biomarkers are elevated donor plasma mitochondrial DNA levels [40], donor urinary C5a levels [41], matrix metalloproteinase-2 levels, periredoxin-2 and periredoxin-1 antitrypsin, and exosomal neutrophil gelatinase-associated lipocalin (NGAL) mRNA that independently predict DGF. Among the recipient biomarkers, cell-free microRNAs (miRNAs) and a short non-coding RNAs that play a pivotal role in regulation of gene expression through epigenetic, transcriptional, and post-transcriptional mechanisms, such as miR-505-3p, have been demonstrated to be an independent predictor of DGF in DCD grafts [42]. Both serum and urine lactate dehydrogenase (LDH) and NGAL have been shown to predict DGF and 1-year graft function, with serum NGAL being more reliable compared to urine NGAL [43,44]. MiRNAs, have been the focus of several studies also in kidney transplant recipients. Recently, a panel of six urine miRNA has been proposed as DGF biomarker as it was found elevated in the first urine voiding after surgery and in urine collected daily in the first days after surgery in patients who developed DGF [45].

Promising biomarkers are also the extracellular vesicles, membrane structures of different size released by cells that could act as mediators of cellular crosstalk between immune system and graft [46]. Some of these, such as plasma endothelial extracellular vesicles, have shown a progressive decrease of their procoagulant activity after kidney transplantation, paralleling with kidney function recovery [47]. Other potential biomarkers have been identified in graft biopsies, such as vimentin and fascin, whose expression on microvasculature appears to be correlate with long-term graft function in patients with DGF [48].

Despite the extensive number of biomarkers that have been put forward over the recent years, still no consensus exists on the utility of any of them for routine clinical practice.

## 2. Therapeutic Approach to AKI in Kidney Transplant

### 2.1. Donor and Recipient-Targeted Therapies

Most therapeutic strategies to prevent DGF experienced so far have targeted the donor before harvesting or the recipient during the peri-operative period. The use of low-dose dopamine for donor pre-treatment before procurement is the strategy that is best supported by evidence coming from clinical trials [49].

The most traditional preventive strategies have been based on preventing graft ischemia by recipient volume expansion before reperfusion with the use of various isotonic saline solutions [50], as well by avoiding preoperative dialysis with subtraction of volume [51]. However, most of the studies targeting the recipients have been based on strategies that reduce the activation of inflammation triggered by adaptive immune response that cause complement activation and endothelial dysfunction [4,22] (Table 2).

Because rabbit anti-thymocyte globulin targets, besides T cells, also endothelial adhesion molecules and may help minimizing CNI use, they have been the induction treatment of choice to prevent DGF. However, no published studies demonstrated a real benefit [52] and the only randomized trial “PREDICT”(NCT02056938) (https://www.clinicaltrials.gov/ct2/show/NCT02056938, accessed on 1 February 2021) specifically designed for this purpose was preliminarily stopped due to poor enrollment.

Encouraging data came from the use of complement inhibitors. Jordan et al. in 2018 preliminarily showed in a phase I/II double blind, placebo-controlled study (NCT02134314, https://clinicaltrials.gov/ct2/show/NCT02134314, accessed on 1 February 2021) that treatment with c1-esterase inhibitor (time 0 and 24 h after transplant) did not reduce DGF incidence but reduced the number and duration of dialysis treatments, with significantly improved kidney function 1 year later [53]. A recent post hoc analysis of the long-term outcomes from this trial showed seven graft failures in the placebo group compared with one among C1 esterase inhibitor-treated recipients. The cumulative incidence of graft failure was lower over 3.5 years among C1 esterase inhibitor-treated recipients compared with placebo, although no difference in eGFR slopes was observed between groups [54]. Anti-C5 antibody (eculizumab) has also been tested to prevent DGF [55], but randomized studies failed to demonstrate clinical efficacy and one study reported an increased incidence of serious adverse events and graft loss in eculizumab-treated patients [55,56].

### 2.2. Organ-Targeted Therapy

The widespread use of machine perfusion for organ storage and of the in situ perfusion of organs from DCD donors have revived interest in treatment strategies aimed at preventing DGF by treating the renal graft ex vivo. Hypothermic perfusion machines are currently the most widely used. They are portable machines that keep low temperature (4–10 °C), deliver a pulsatile flow, and optionally provide oxygenation (oxygenated versus non-oxygenated machine perfusions). A meta-analysis has shown that hypothermic machine perfusion is superior to static cold storage in preventing DGF in deceased donor kidney transplantation [57]. This is true for both DBD and DCD kidneys. As kidneys from DCD donors have a higher overall DGF rate, fewer perfusions are needed to prevent one episode of DGF (7 vs. 14 in DBD kidneys) [57]. However, in the hypothermic state, cells from the graft have dampened metabolic activity. Interest has grown around normothermic machine perfusion, in which the perfusion temperature is between 35 and 37 °C [58]. This technique of organ preservation facilitates restoration of cellular metabolism, reviving the organ ex vivo, which eventually resumes its normal physiological functions [59]. Therefore, theoretically, normothermic machine perfusion enables the graft to respond to pharmacological treatment.

Among the most promising pharmacological treatments that may be administered to the graft via machine perfusion to prevent DGF and fibrosis is cell therapy based on multipotent adult progenitor cells (MAPC) [60]. Multipotent adult progenitor cells possess immunomodulatory properties which could prove beneficial in minimizing subsequent ischemia reperfusion injury. Thompson et al. investigated the potential reconditioning capability of ex vivo administration of MAPC in a pre-clinical normothermic machine perfusion model in kidney [61]. MAPC-treated kidneys showed improvement in urine output, decreased expression of the kidney injury biomarker NGAL, and improved microvascular perfusion [61].

One additional strategy is the complement inhibitor APT070 (Mirococept) with a unique ‘cytotopic’ property that permits its retention in the organ microvasculature. This latter drug is currently being investigated in the EMPIRIKAL trial for the prevention of DGF in deceased-donor kidney transplantation [62].

## 3. Early AKI in the Transplant Recipient after Initial Recovery

AKI might develop early after transplantation after an initial recovery of graft function. The most common causes are surgical or medical complication, including acute rejection, whose diagnosis requires biopsy. Acute tubular necrosis, in the absence of inflammatory injury, is reported as one of the possible phenotypes of antibody-mediated rejection [63]. Surgical (either vascular or urological) complications can be easily identified by renal ultrasound and/or CT scan. At this stage, ischemic acute tubular necrosis may be secondary to correctable causes such as hypovolemic status, high blood level of calcineurin inhibitors, arterial hypotension, and infections complicating the post-surgical course. Rarely, however, AKI may be the result of occult systemic disease that remained unrecognized until transplantation, and that manifest as disease recurrence in the graft. Among them, acute crystal nephropathy may be caused by recurrent 2,8-dihydroxyadenine nephropathy, which may lead to graft loss if left untreated [64]. Primary or secondary hyperoxaluria (e.g., in obese patients undergoing malabsorptive surgery before transplantation) may be an additional cause of acute crystal nephropathy [65].

## 4. Long-Term AKI in the Transplant Recipient

AKI after transplantation is a risk factor for graft failure [66,67]. Most common causes of AKI in the long-term follow-up post-transplantation can be divided into two groups: (1) asymptomatic AKI and (2) AKI with systemic symptoms. Asymptomatic AKI may be caused by acute rejection in patients with poor drug adherence, or by polyomavirus BK infection nephropathy. However, it is most commonly secondary to drug toxicity and drug-to-drug interaction with calcineurin inhibitors (CNI) [68,69]. Non-steroid anti-inflammatory drugs (NSAID) are a common cause of AKI from nephrotoxic drugs. Recently, one long-term longitudinal cohort study assessed risk of AKI with NSAID prescriptions in kidney transplant recipients [70]. NSAID prescriptions were dispensed to 5% of kidney transplant recipients (2 per 100 patient-years), with 70% of them receiving high doses [70]. The median time from transplant to NSAID prescription was 4 years (interquartile range: 2–5 years). NSAID prescription was associated with a significantly increased risk of AKI, which was further augmented by higher NSAID dose and longer NSAID duration [70].

Among the cases of AKI with symptoms, bacterial infection and especially urinary tract infections are by far the most common cause [71]. The prevalence of urinary tract infection history among kidney transplant recipients varies widely from 23% to 75%, with associated bacteremia occurring in 40% of them [71]. The highest incidence has been reported in the first 3–6 months [71]. Infection with hypovolemia is an additional common cause, which occurs in the setting of acute gastroenteritis causing vomiting and diarrhea.

Garg et al. recently analyzed trends in rates of hospitalizations in kidney transplant recipients with primary diagnosis of AKI, secondary diagnosis of AKI, and AKI-requiring dialysis over an 11-year study period [72]. Incidence of hospitalization increased in most recent eras for all types of AKI, mostly driven by sepsis. Overall, risk of hospitalization for AKI was 5% (11% in the first 3 years). Primary AKI hospitalization rate showed an annual increase of +7.0%, and secondary AKI an annual increase rate of 21%. However, the increasing trend of hospitalization for AKI may reflect increasing diagnosis of milder forms of AKI in less sick patients occurred in most recent years.

An automated real-time electronic (e)-alert system for AKI, based on the Kidney Disease Improving Global Outcomes (KDIGO) change in creatinine diagnostic criteria, was developed in England and Wales in order to identify AKI in the general population and facilitate a better outcome [73,74]. Recently, this system was also applied in the transplant population by an automated biochemistry-based electronic AKI alert [66]. A prospective national cohort study, which collected data on 1224 renal transplants recipients (2010–2014), showed that 35% patients had at least one episode of AKI [66]. This incidence rate was higher than the 12% reported by Mehrotra et al. [75], probably because it involved also non-hospitalized patients.

Because the evidence that these alerts impact outcome is lacking, further developments of those electronic alerts and clinical studies are needed before implementation can be recommended for routine clinical care.

## 5. COVID-19-Associated AKI in Kidney Transplant Recipients

In December 2019, a novel strain of Coronavirus, severe acute respiratory syndrome coronavirus 2 (SARS-CoV-2), was identified as the causative agent in a cluster of patients presenting with severe pneumonia in Wuhan, China. Multiple reports converged to indicate that kidney transplant recipients have higher mortality than the general population, possibly because of the ongoing immunosuppression and multiple co-morbidities [76,77]. AKI is a common finding in patients with coronavirus disease 2019 (COVID-19), possibly due to direct and indirect viral injury, and has been associated with higher rates of death when compared to COVID-19 patients without AKI [76,78,79]. Although graft biopsies have not been performed routinely, AKI in kidney transplant recipients with COVID-19 does not seem to be commonly due to acute rejection, despite the frequent reduction of antirejection therapy during infection [76].

## 6. Conclusions

AKI, a common event in kidney transplantation in both the donor and the recipient, may have consequences on both short- and long-term graft functions. Several studies have been performed trying to identify biomarkers for predicting which donor AKI carries the highest risk of graft failure, and to implement treatment strategies to minimize the impact of AKI on short- and long-term graft dysfunction. While the research on biomarkers has not translated into clinical applications, the use of hypothermic machine perfusion has become a consolidated practice to prevent DGF. AKI occurring after early post-transplantation after initial recovery of graft function is usually related to surgical or medical causes that may occasionally cause graft failure if left untreated. Development of AKI later after transplantation often has an unfavorable impact on allograft outcomes because it may reflect the chronic use of nephrotoxic drugs or serious underlying medical conditions. E-alert systems have been proposed to help in identifying outpatients developing AKI during long term follow-up, but evidence on the beneficial effect on patient outcomes is still lacking. Longer follow-up studies are needed to understand the impact of COVID-19-assoaicted AKI in kidney transplant recipients.

## Figures and Tables

**Table 1:** The most common causes of DGF.

**Donor-Related Risk Factors**
AKI and hemodynamic instability in ICU
Prolonged cold ischemia time
Graft quality (old age, CKD risk factors)
Donor type (DCD vs. DBD vs. living donor)
**Recipient-Related Risk Factors**
Surgery
Complex vascular surgery/vascular complications (prolonged warm ischemia time)
Increased BMI, concomitant surgery (e.g., ADPKD nephrectomy)
High immunological risk/rejection
Pre-transplantation oliguria (HD vs. PD; long dialysis vintage vs. pre-emptive)
Pre-transplantation HD/UF session
**Perioperative Risk Factors**
Peri-operative hypotension/hypovolemia
High CNI blood levels

Abbreviations: DGF, delayed graft function; ICU, intensive care unit; CKD; chronic kidney disease; DCD, donation after circulatory death; DBD, donation after brain death; BMI, body mass index; ADPKD, adult dominant polycystic kidney disease; HD, hemodialysis; PD, peritoneal dialysis; UF, ultrafiltration; CNI, calcineurin inhibitors.

**Table 2:** Newer recipient-oriented therapies for AKI.

Injury and Inflammation in Mediating AKI
Thymoglobulin/CNI-sparing regimens—“PREDICT” trial, NCT02056938 (https://www.clinicaltrials.gov/ct2/show/NCT02056938, accessed on 1 February 2021)
Inhaled carbon monoxide
Recombinant P and E selectin ligand
Anti-intracellular adhesion molecule 1 antibody
Complement inhibitors
C1-esterase inhibitor
Anti-C5 antibody—eculizumab, “PROTECT” trial, NCT02145182 (https://clinicaltrials.gov/ct2/show/NCT02145182, accessed on 1 February 2021)
Cell death and protective factors in mediating AKI and recovery
Hepatocyte growth factor—NCT02474667, and refanalin IV (https://clinicaltrials.gov/ct2/show/NCT02474667, accessed on 1 February 2021)
Diannexin—NCT01442337 (https://clinicaltrials.gov/ct2/show/NCT01442337, accessed on 1 February 2021)
siRNA-targeting p53 (QP-1002)—“ReGIFT” trial; NCT02610296 (https://clinicaltrials.gov/ct2/show/NCT02610296, accessed on 1 February 2021)

AKI, acute kidney injury; CNI, calcineurin-inhibitors; C1, complement 1 (Mannon et al. [22]).

## Abbreviations

AKI	Acute kidney injury
BMI	Body mass index
CIT	Cold ischemia time
CKD	Chronic kidney disease
DAMP	Damage-associated molecular
DBD	Donation after brain death
DCD	Donation after circulatory death
DGF	Delayed graft function
ECD	Extended criteria donor
GFR	Glomerular filtration rate
HIF	Hypoxia-inducible factor
KDIGO	Kidney Disease: Improving Global
KPDI	Kidney donor profile index
MAPC	Multipotent adult progenitor cells
NGAL	Neutrophil gelatinase-associated
PAMP	Pathogen-associated molecular pa
PNF	Primary non-function
S1P	Sphingosine-1-phosphate receptor
TLR	Toll-like receptors
